# Coupling of Voltage-Sensors to the Channel Pore: A Comparative View

**DOI:** 10.3389/fphar.2012.00145

**Published:** 2012-07-27

**Authors:** Vitya Vardanyan, Olaf Pongs

**Affiliations:** ^1^Ion Channel Research Group, Institute of Molecular Biology, National Academy of Sciences of the Republic of ArmeniaYerevan, Armenia; ^2^Zentrum für Molekulare Neurobiologie HamburgHamburg, Germany; ^3^Physiologisches Institut, Universität des SaarlandesHomburg, Germany

**Keywords:** ion channels, voltage-sensors, electromechanical coupling

## Abstract

The activation of voltage-dependent ion channels is initiated by potential-induced conformational rearrangements in the voltage-sensor domains that propagates to the pore domain (PD) and finally opens the ion conduction pathway. In potassium channels voltage-sensors are covalently linked to the pore via S4–S5 linkers at the cytoplasmic site of the PD. Transformation of membrane electric energy into the mechanical work required for the opening or closing of the channel pore is achieved through an electromechanical coupling mechanism, which involves local interaction between residues in S4–S5 linker and pore-forming alpha helices. In this review we discuss present knowledge and open questions related to the electromechanical coupling mechanism in most intensively studied voltage-gated *Shaker* potassium channel and compare structure-functional aspects of coupling with those observed in distantly related ion channels. We focus particularly on the role of electromechanical coupling in modulation of the constitutive conductance of ion channels.

## Introduction

Voltage-dependent ion channels are important components of excitable biological membranes responsible for initiation, propagation, and shaping of action potentials (Hille, 2001). In non-excitable cells they are in charge of electrolyte transport into and out of cells. Numerous gene mutations of ion channels are implicated in human inherited diseases, pathologies of which are often associated with impaired voltage control of channels (Ashcroft, 1999). Discovery of new therapeutic agents targeting ion channels requires a solid knowledge of structure-function correlation of these complex membrane proteins.

In recent decades the molecular mechanisms of permeation, selectivity and conductance in many ion channels have been revealed with unprecedented precision by combination of electrophysiological, biochemical, and crystallographic methods. In this respect, potassium (K) channels are the best understood ion channels, which are integral membrane proteins typically formed by the association of four identical subunits (Doyle et al., 1998). The Pore Domain (PD) of K channels represents a tetramer of two membrane-spanning alpha helixes that are connected with each other via a P-loop, which is responsible for potassium selectivity. The PD contains a channel gate, which controls ion permeation (Holmgren et al., 1998; del Camino and Yellen, 2001; Swartz, 2004; Webster et al., 2004; del Camino et al., 2005). The structural correlate of the gate is a bundle of overcrossing alpha helixes at the cytoplasmatic entryway of the channel pore (Holmgren et al., 1998). These alpha helices correspond to the M2 helix of the non-voltage-gated (Doyle et al., 1998) and to the S6 helix of voltage-gated potassium (Kv) channels. To close the gate, the pore constricts and forms a hydrophobic seal by side-chains of converging S6 pore helices (Doyle et al., 1998; Holmgren et al., 1998; del Camino and Yellen, 2001; Hackos et al., 2002). Opening and closing of Kv channel gate is under the stringent control of the membrane electric potential (Islas and Sigworth, 1999). In Kv channels the pore is covalently linked to four specialized membrane-embedded peripheral functional modules, Voltage Sensing Domains (VSDs) that are comprised of S1–S4 membrane-spanning segments. VSDs are capable of sensing changes in membrane potential and respond with conformational changes that propagate to the PD (Bezanilla, 2000; Jiang et al., 2003; Catterall, 2010). Available crystal structures along with functional data suggest that VSDs are semi-independent functional units (Long et al., 2005a, 2007; Alabi et al., 2007). In recent years, a significant progress has been made toward the elucidation of the molecular architecture and functional principles of these specialized domains (Bezanilla, 2008; Catterall, 2010; Tao et al., 2010). VSDs are connected to the PD by a linker, known as S4–S5 linker at the cytoplasmic side of the membrane. The VSD-PD assembly represents an exquisite molecular electromechanical coupling device, which converts potential energy of the membrane electric field into the mechanical work needed to control the selective permeation of potassium ions. Several functional studies indicate that concordance between the S4–S5 linker and distal S6 region of the PD is important for transmission of conformational changes from VSDs to PD (Chen et al., 2001; Lu et al., 2001, 2002; Decher et al., 2004; Long et al., 2005b; Soler-Llavina et al., 2006; Labro et al., 2008; Lee et al., 2009; Choveau et al., 2011). How the conformational changes originating in VSDs are transferred to the PD and how they influence the functional state of the channel gate remains an unresolved issue.

In this review we summarize current knowledge and open questions concerning to the molecular and structural aspects of electromechanical coupling. The *Shaker* Kv channel is the most intensively investigated voltage-gated channel in terms of electromechanical coupling. Here, we highlight similarities and key differences of electromechanical coupling process in *Shaker* and in some distantly related ion channels.

## Current View of Electromechanical Coupling

It is well established that voltage-sensors are semi-independent membrane-embedded modules (Alabi et al., 2007; Long et al., 2007) that undergo conformational changes upon alterations of membrane potential (Bezanilla, 2000, 2008; Villalba-Galea et al., 2008). The details of conformational changes that actually take place in VSDs are a subject of much debate (Campos et al., 2007; Borjesson and Elinder, 2008; Broomand and Elinder, 2008; Villalba-Galea et al., 2008; Tao et al., 2010; Vargas et al., 2011). It is not completely understood how and to which extent positive charges of S4 helix move upon a change in membrane potential (Campos et al., 2007; Bezanilla, 2008). It is also unclear how homogeneous is the electric field that membrane-embedded VSDs experience (Bezanilla, 2008; Catterall, 2010).

Transmission of conformational changes from each voltage-sensors to the pore was thought to occur primarily through S4–S5 linker, since the latter directly connects dynamic S4 segment of the VSDs to S5 segment of the PD. A decade ago Lu et al. (2002) first demonstrated that interaction between S4–S5 linker and distal S6 segment of the PD is also essential for coupling. The molecular and structural aspects of this phenomenon remained obscure until crystal structures of *Shaker*-related channels were resolved with remarkable resolution (Long et al., 2005a,b, 2007). Two non-covalent interaction interfaces between VSDs and PD were observed in crystal structures of *Shaker*-related channels. An extensive interface was observed at the cytoplasmatic side of the membrane involving side-chains of S4–S5 linker and side-chains of the distal S6 segment (Long et al., 2005b, 2007). The second, comparably small interface was found at the extracellular side of the pore formed by interaction of side-chains of S1 segment of VSDs with side-chains of S5 in the pore (Long et al., 2005a, 2007; Lee et al., 2009). It has been suggested that latter interface plays a role in effective translocation of charged S4 helix during membrane voltage changes (Lee et al., 2009). An essential role for transmission of conformational changes from VSDs to the channel gate was given to the interactions between S4–S5 linker and distal S6 (Labro et al., 2005; Long et al., 2005b).

S4–S5 linkers in *Shaker*-related channels adopt an amphipathic alpha helical conformation (Long et al., 2005a, 2007) both in crystals and in solution (Ohlenschlager et al., 2002). The functional mutagenesis data suggest that changes in the amphipathic configuration of S4–S5 helix disturb the electromechanical coupling process (Labro et al., 2008). In the activated Kv channel, the S4–S5 helix runs parallel to the intracellular side of the lipid bilayer (Long et al., 2005b, 2007) with its hydrophobic side facing the lipid membrane. The hydrophilic face of S4–S5 linker is exposed to the cytoplasm (Long et al., 2007). Currently, it is unknown whether the hydrophilic side is involved in specific interactions with other parts of the ion channel. The interaction between S6 helix with its “receptor” site on the S4–S5 linker requires the pore helices to bend or kink (Long et al., 2005b). Most likely, this is associated with the occurrence of a conserved PXP motif and a “gating hinge” glycine in S6 helix (Ding et al., 2005; Long et al., 2005b). The molecular interactions between the side-chains of S4–S5 helix and side-chains of the distal S6 segment (Long et al., 2005b) apparently play a key role for transmission of conformational changes during channel gating. It is likely that interaction between S4–S5 linker and distal S6 takes place also at the closed state of the channel. Molecular dynamic simulations studies predict the movement pattern of S4–S5 linker as well as its interaction with S6 during the voltage gating process of *Shaker*-related channels (Yarov-Yarovoy et al., 2006; Jensen et al., 2012). Summarizing experimental and theoretical studies Blunck and Batulan discuss two alternative scenarios where S4–S5 linker functions (1) as a spring and (2) as a bolt (see current review issue).

In spite of available nice models, the essential questions need to be addressed experimentally in order to understand the electromechanical coupling mechanism in detail. For example, which channel states are predominantly influenced by interaction of S4–S5 linker and S6? Are these interactions the only determinants of electromechanical coupling or other factors (interactions) play a significant role (see for example Lee et al., 2009; Batulan et al., 2010)? Do similar molecular and structural principles govern electromechanical coupling in ion channels distantly related to *Shaker*? Of note is that mutagenesis experiments have shown that the interactions between S4–S5 linker and the distal S6 play a key role in electromechanical coupling also in hERG- (Ferrer et al., 2006), KCNQ1- (Choveau et al., 2011), HCN- (Chen et al., 2001), and KAT1-channels (Ferrer et al., 2006; Grabe et al., 2007). In Kv4 channels coupling strength determines the inactivation properties of channels (Barghaan and Bahring, 2009; Bahring et al., 2012). Nevertheless, electrophysiological analysis of S4–S5 linker mutants revealed some essential differences between *Shaker* and more distantly related channels that we will describe below. Elucidation of these differences will help us to further our knowledge about the electromechanical coupling process and design new comprehensive studies in future.

## Uncoupling Voltage-Sensors from the Pore in *Shaker*

**Figure 1:**
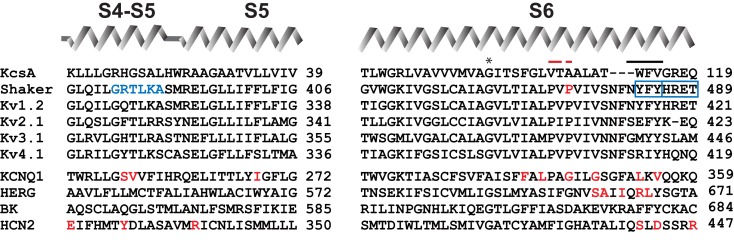
**Sequence alignment of relevant Kv channels in the S4–S5 linker and in S6 segment**. Diagram above the sequences represents secondary structure elements corresponding to the recent crystal structure of Kv1.2–Kv2.1 chimeric channel in lipid-like environment (Long et al., 2007). Residues indicated by red letters show localization of the mutations involved in constitutive conductance of corresponding channels. Residues shown with blue letters and blue frames indicate position of mutations, which render *Shaker-*KcsA chimeras (carrying distal S6 starting from I477, as well as S4–S5 linker from *Shaker*, but the rest of the pore from KcaA) non-functional (Lu et al., 2002). Red bar indicates the position of PXP motive of Kv channels; black bar indicates localization of the gate. Asterisk shows gating hinge glycine. Sequence identification numbers are as follows: KcsA, GI 612269090; *Shaker*, GI 288442; rat Kv1.2, GI 24418849; human Kv2.1, GI 4826784; human Kv3.1, GI 163792201; human Kv4.1, GI 27436981; human KCNQ1, GI 32479527; mouse HCN2, GI 6680189; hERG, GI 4156239; rat BK, GI 13929184.

In voltage-dependent ion channels the effectiveness of converting electrical energy into mechanical work that influences the state of the channel gate is dependent on the strength of electromechanical coupling. If the coupling is strong, the gate will be under the tight control of VSDs. If the electromechanical coupling between VSDs and the PD is markedly weaker or absent, the conformational changes in VSDs will no longer be able to influence the opening or the closing of channel gate. Mutations or chimeric replacements localized in S4–S5 linker and/or S6 region significantly affect the gating properties of voltage-gated ion channels (Sullivan et al., 1997; Lu et al., 2001, 2002; Tristani-Firouzi et al., 2002; Ferrer et al., 2006; Labro et al., 2008; Barghaan and Bahring, 2009; Haddad and Blunck, 2011). Moreover, a fractional voltage-dependent and voltage-independent conductance was observed in some mutants, indicating that modulation of the coupling strength can markedly influence also the stability of the resting-closed state of channels. In Figure 1 we have highlighted the localization of mutations having a drastic influence on the functional properties of channels.

The first comprehensive mutagenesis studies, focusing on the molecular aspects of the electromechanical coupling were started by Lu et al. (2001, 2002). The results indicated that two large segments, one corresponding to the S4–S5 linker and the other to the distal part of S6, should be preserved in *Shaker* in order to maintain full voltage control over *Shaker* channel gating (Lu et al., 2002). Mismatches between S4–S5 linker and distal S6 by replacement of corresponding segments of *Shaker* by KcsA resulted in channels with a partial constitutive conductance. Single channel analysis revealed that the constitutive conductance in these channels reflects spontaneous fluctuations of the channel between open and closed states (Lu et al., 2002) at resting voltage-sensor conditions. In wild type *Shaker* channels spontaneous channel openings at hyperpolarized potentials are rare events with an estimated open probability of 10^−9^ (Islas and Sigworth, 1999). Results indicated that the constitutive conductance in *Shaker*-KcsA chimeras can be explained by a weakening of coupling between VSDs and PD. Hydrophilic substitutions at residue Pro475 in *Shaker* also caused a constitutive conductance, in spite of the fact that P475 is located within the conduction pathway of the channel (Hackos et al., 2002; Sukhareva et al., 2003). Thus, it has been proposed that coupling strength plays a significant role for constitutive conductance in P475 hydrophilic mutants too (Sukhareva et al., 2003).

Haddad and Blunck (2011) recently proposed an alternative explanation of uncoupling in *Shaker* studying the mode shift of gating charge translocation in VSDs (Haddad and Blunck, 2011). They suggest that a marked reduction in coupling strength, or complete uncoupling, will result in permanently closed *Shaker* channels. This proposal was based on thorough electrophysiological analysis of S4 segment translocation and voltage-dependent conductance of two *Shaker* mutations I384N and F484G (Haddad and Blunck, 2011). The choice of the mutations was based on recent crystal structures of Kv channels (Long et al., 2005b, 2007). One mutation was localized at S4–S5 linker (I384N) and the other within distal S6 (F484G). Uncoupling in these two mutants was characterized by a significant leftward shift in the gating charge (Q) – voltage (V) relation (QV curve) accompanied with rightward shift in conductance (G) – voltage (V) relationship (GV curve). A shift of QV curve to more negative potentials indicates that less energy is required for translocation of VSDs from a resting to an activated position in mutant. I384N and F484G *Shaker* channels were expressed robustly on plasma membrane but only a small fraction of channels could be opened even by extreme depolarization pulses (Haddad and Blunck, 2011). This indicates that the channel gate remained mostly closed although voltage-sensors could be easily translocated from the resting state to an activated state. Conversely, I384A *Shaker* mutation considerably detained the voltage-sensor movement judged by a large positive shift in the QV curve. Taking into consideration that the GV relation is almost unchanged in I384A, authors suggested that mutant channels display a stronger coupling than wild type (Haddad and Blunck, 2011). In summary, it was suggested that at the resting state the *Shaker* pore applies a mechanical load onto VSDs. The uncoupling mutations I384N and F484G take this mechanical load off, thereby facilitating the translocation of gating charges, but arresting the channel pore in a closed state. Strengthening of coupling between VSDs and the pore in the I348A mutant constrains S4 segment translocation but facilitates channel opening. Similar considerations were made in earlier studies regarding to the gating current kinetics of F484 *Shaker* mutants (Ding and Horn, 2003).

At first glance, abovementioned two explanations for uncoupling in *Shaker* seem somewhat contradictory. The first states that uncoupling in *Shaker* promotes the formation of channels with constitutively open pore, whereas the second hypothesis proposes that *Shaker* channels with uncoupled voltage-sensors are permanently closed. Analysis of voltage-sensor movement and plasma membrane expression of constitutively conducting *Shaker*-KcsA channels may shed a light on this ambiguity.

## Electromechanical Coupling in Channels Distantly Related to *Shaker*

Principles of electromechanical coupling were elucidated on the basis of functional and structural studies carried out predominantly on *Shaker* or related potassium channels. To substantiate the generality of these principles, the electromechanical coupling was investigated also in channels distantly related to *Shaker*, e.g., KCNQ1, HERG, and HCN2 channels (Sanguinetti and Xu, 1999; Chen et al., 2001; Tristani-Firouzi et al., 2002; Decher et al., 2004; Macri and Accili, 2004; Ferrer et al., 2006; Prole and Yellen, 2006; Boulet et al., 2007; Labro et al., 2008, 2011; Choveau et al., 2011). The low sequence homology of these channels with *Shaker* makes it difficult to correlate the mutational data with currently available structural models of K channels (Long et al., 2005a, 2007; Jensen et al., 2012). In spite of low sequence homology, it is remarkable that gating properties of KCNQ1, HERG, and HCN2 channels change similarly when mutating the corresponding S4–S5 linker and distal S6 regions (Figure 1). Electrophysiological analysis of KCNQ1, HERG, and HCN2 channels carrying mutations or chimeric replacements in S4–S5 linker and S6 showed three main phenotypes: (1) GV relations exhibit significant shifts to both positive and negative potentials; (2) some mutants were non-functional in heterologous expression systems; (3) a significant number of point mutants gave rise to constitutively conducting channels. Constitutive conductance in *Shaker* was observed only upon substitution of P475 pore residue with amino acids having hydrophilic side-chains, which we will discuss below in detail. At this point, we will briefly summarize the main findings of studies related to the electromechanical coupling in KCNQ1, HERG, and HCN2 channels.

Exchange of the entire S6 segment of hERG potassium channel with its counterpart from bovine ether-a-go-go (bEAG) resulted in hERG/bEAG chimeric channel with a partial constitutive conductance (Ficker et al., 1998). In order to restore complete voltage-gated features of the chimeric channel, it was necessary to reconstitute the distal S6 hERG sequence (SAIIQRL) in the hERG/bEAG chimera. Furthermore, transfer of bEAG S4–S5 linker to the hERG disrupted the complete hERG channel closure (Ferrer et al., 2006). Substitution of distal S6 region with corresponding bEAG segment in this chimera fully restored the channel closure at hyperpolarized potentials (Ferrer et al., 2006). Further mutational analysis showed that an exchange of five amino acids (colored red in Figure 1) sufficed to restore fully voltage-gated properties. These results accentuate the concordance between S4–S5 linker and S6 sequences for complete closure of hERG channel at hyperpolarized potentials (Ferrer et al., 2006). Data are also in agreement with an earlier suggestion that S4–S5 linker and distal S6 in HCN2 channel are in close proximity at hyperpolarized membrane potentials (Tristani-Firouzi et al., 2002). This proposal was based on electrophysiological analysis of channels carrying mutations at D540 (S4–S5 linker) and R665 (S6) residues.

Recent scanning mutagenesis of KCNQ1 channel revealed that S4–S5 linker and distal S6 are the most sensitive regions for mutational gating perturbations (Ma et al., 2011). A significant number of mutants in these regions drastically affected KCNQ1 channel function by shifting GV relations toward positive and, respectively, negative directions. Boulet et al. (2007) and Choveau et al. (2011) obtained similar results for KCNQ1 channel. Moreover, analysis of double-mutant, constructed by combination of two point mutants, one located on S4–S5 linker (V254A), the other on S6 (L353A), confirmed the hypothesis that S4–S5 linker and S6 interact with each other to ensure the voltage-dependent gating of KCNQ1 (Choveau et al., 2011). This interaction, however, appears to be state-dependent, i.e., it only takes place in the closed state of the channel (Choveau et al., 2011).

Electromechanical coupling is well investigated also in hyperpolarization-activated cyclic nucleotide gated cation (HCN) channels. HCN channels are particularly interesting from this point of view, since they have an inverse voltage-dependent gating mode, i.e., hyperpolarization activates, whereas depolarization deactivates HCN channels (Brown et al., 1979; Gauss et al., 1998; Biel et al., 2009). Alanine scanning mutagenesis of the S4–S5 linker region in HCN2 revealed several amino acid residues, mutation of which gave rise to a channel with partial constitutive conductance (Chen et al., 2001). Mutations causing a constitutively open HCN2 channel were also identified in its distal S6 region (Decher et al., 2004). Based on the functional analysis of double mutants, it has been proposed that electrostatic interaction between side-chains of S4–S5 linker and distal S6 mediate electromechanical coupling in HCN2 channels (Decher et al., 2004). The coupling, however, is thought to be completely disrupted at depolarized potentials which leads to the closure of channel gate also known as voltage desensitization (Shin et al., 2004).

## Evaluating the Similarities and Differences

A significant number of mutations in S4–S5 linker and distal S6 markedly alter the gating properties of *Shaker* and related channels (Isacoff et al., 1991; McCormack et al., 1991; Schoppa et al., 1992; Li-Smerin et al., 2000; Hackos et al., 2002; Yifrach and MacKinnon, 2002; Ding and Horn, 2003; Soler-Llavina et al., 2006). Similar observations have been made upon mutations of the corresponding regions of KCNQ1, hERG, and HCN2 channels (Donger et al., 1997; Chen et al., 2000; Decher et al., 2004; Ferrer et al., 2006; Boulet et al., 2007; Ma et al., 2011). Yet, there are noteworthy differences. Examination of available mutational data revealed that point mutations rarely cause a constitutive conductance in *Shaker* (Yifrach and MacKinnon, 2002; Soler-Llavina et al., 2006). By contrast, analysis of KCNQ1 and HCN2 channels carrying comparable point mutations in their S4–S5 linker and distal S6 regions showed that quite a significant number of mutant KCNQ1 and HCN2 channels demonstrate a large constitutively open component (Chen et al., 2001; Decher et al., 2004; Ma et al., 2011). These data indicate that KCNQ1, HCN2, and hERG channels are more susceptible to constitutive conductance than the *Shaker* channel.

Two different mechanisms are discussed in literature as possible cause of partial or complete constitutive conductance in voltage-gated ion channels. A leak of the ions through the channel pore due to incomplete closure of the gate at resting voltage-sensor conditions is one possible mechanism. Single channel measurements of mutant *Shaker* channels (Islas and Sigworth, 1999; Sukhareva et al., 2003) as well as BK channel (Horrigan and Aldrich, 2002) indicated that the pore is able to completely prevent the ion flow. This suggests that the constitutively open component of conductance is due to spontaneous fluctuations of channel between the open and the closed states in resting voltage-sensor conditions (Horrigan and Aldrich, 2002; Sukhareva et al., 2003; Niu et al., 2004). Thus, the resting sate of KCNQ1 as well as HCN2 channels allows significant spontaneous transitions to the open state independent of changes in membrane voltage (Decher et al., 2004; Ma et al., 2011). Significant number of point mutations in S4–S5 linker and distal S6 in these two channels even more destabilized the resting-closed state, in exceptional cases leading to constitutively open phenotype.

Evaluating the energetics of pore opening by a double-mutant cycle analysis, it has been proposed that the *Shaker* pore is intrinsically closed, i.e., the closed state is energetically favored in virtual absence of VSDs (Yifrach and MacKinnon, 2002). Analysis of *Shaker* mutants with respect to gating charge movement (Ding and Horn, 2003) and mode shift in VSDs translocation (Haddad and Blunck, 2011) were in agreement with this conclusion. Considering VSDs and PD as two separate elements of the VSD-PD bimodular system, in current issue Blunck and Batulan describe the electromechanical coupling from thermodynamic point of view. Analysis suggests that strong electromechanical coupling will require less energy for opening of the channel pore with a given probability. It follows, that complete uncoupling of the gate from VSDs will lead to permanently closed channels, since energy provided by VSDs will no longer reach to the PD.

If channel possesses an intrinsically open pore, uncoupling would lead to constitutively open channels. In these channels, VSDs must exert a mechanical work to close the pore. This hypothesis reasons that various degrees of constitutive conductance in KCNQ1, HCN2, and hERG mutants are caused by alteration in coupling strength. In extreme cases, when coupling between VSDs and PD is lost, channels open constitutively as observed in some mutant KCNQ1 and HCN2 channels (Chen et al., 2000; Ma et al., 2011). Nevertheless, *Shaker* hydrophobic mutations at P475 position (Hackos et al., 2002; Sukhareva et al., 2003) and *Shaker*-KcsA chimeras (Lu et al., 2001, 2002) also show various degrees of constitutive conductance. How can these results be reconciled with our hypothesis?

P475 position in *Shaker* corresponds to the second proline of the PXP motif of K channel pores. The PXP motif may provide flexibility to the inner S6 helixes in most of Kv channels (del Camino et al., 2000) and allow the bending of S6 helixes at the cytoplasmic side of the pore (del Camino and Yellen, 2001). Studies investigating the constitutive conductance in *Shaker* suggested that hydrophilic substitutions at P475 position change the electromechanical coupling between VSDs and the gate (Hackos et al., 2002; Sukhareva et al., 2003). P475 is localized in the pore of the *Shaker* channel, quite far away from the putative coupling interface proposed by MacKinnon and colleagues (Long et al., 2005b). Recent crystal structures indicate that the bending of the S6 helices makes the interaction between S4–S5 linker helix and distal S6 region topologically possible (Long et al., 2005b, 2007). Thus, one possible explanation is, that the hydrophilic P475 mutations change the electromechanical coupling strength by influencing the bending properties of pore helixes. Since other P475 substitutions, which also influence the flexibility of S6 helices, do not cause a constitutive conductance (Li-Smerin et al., 2000; Sukhareva et al., 2003; Soler-Llavina et al., 2006), bending of S6 seems insufficient to explain the data. Significant changes in voltage gating properties observed in P457 hydrophilic mutants hint toward a more complex picture of mutational influence (Sukhareva et al., 2003). Weakening the strength of electromechanical coupling accompanied by alteration in intrinsic pore properties it the likely cause of partial constitutive conductance phenotype in *Shaker* P475 hydrophilic mutants.

**Figure 2:**
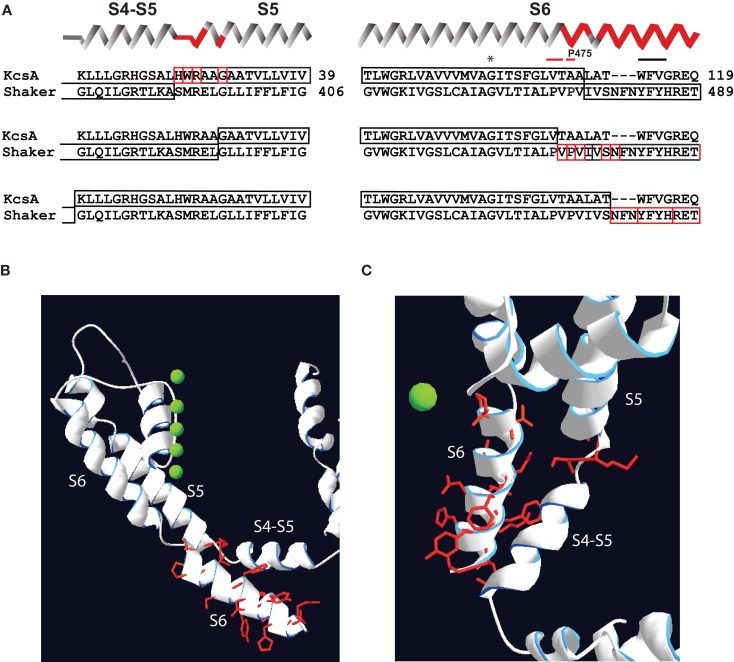
**Localization of mutations susceptible for constitutive conductance in *Shaker*-KcsA chimeric channels according to recent Kv crystals**. **(A)** Three groups of KcsA-*Shaker* chimeras that demonstrated partial or almost complete constitutive activation are shown separately. Secondary structure succession pattern on the top of sequence alignments corresponds to the recent crystal structure of the Kv1.2–Kv2.1 chimera (Long et al., 2007). Red-framed residues indicate the extension of the chimeric link (one residue at a time) causing constitutive conductance. **(B)** Localization of residues susceptible for constitutive conductance mapped on crystal structure of Kv1.2–Kv2.1. The side-chains of these residues are represented as red sticks. Only one subunit is shown for simplicity. **(C)** Top view of same structure shown in **(B)**.

We reexamined the results of Shaker-KcsA chimeric replacements studied by Lu et al. (2002) in the light of the structural data that became available after the work was published (Long et al., 2005a, 2007). According to our analysis the mutational manipulations causing a partial constitutive conductance in *Shaker*-KcsA and Kv2.1-KcsA chimeras (Lu et al., 2002) are located in proximal S5 (SMRELGL sequence in *Shake*r) and in distal S6 (PVPVIVSN sequence in *Shaker*; Figure 2). Careful assessment also indicates that mutations restricted to S4–S5 linker are not able to produce constitutively open channels. Apparently, changes outside of the S4–S5 linker are required to generate constitutively open *Shaker* channels.

The distal S6 segments of KscA and *Shaker* channels differ significantly in sequence as well as in structural organization (Doyle et al., 1998; Long et al., 2007). Therefore, it is possible that chimeric replacements of *Shaker* S6 segment with the corresponding KcsA sequence (Lu et al., 2002) markedly influence the intrinsic properties of the *Shaker* pore. Thus, we propose that *Shaker*-KcsA chimeras and P475 hydrophilic substitutions affect two important features in *Shaker*: (1) coupling strength between VSDs and the PD is decreased and (2) intrinsic properties of pore are altered.

It follows that the pores of wild type KCNQ1 and HCN2 channels are likely to be thermodynamically more stable at their open state in the absence of the voltage-sensors, i.e., intrinsically more stable at the opened state. Two important features observed in these channels corroborate this notion. (1) Wild type KCNQ1 and HCN2 channels demonstrate a significant constitutive conductance at resting voltage-sensor conditions (Decher et al., 2004; Ma et al., 2011). (2) A large number of S4–S5 linker mutants significantly alter the constitutively open component in these channels (Chen et al., 2001; Decher et al., 2004; Boulet et al., 2007; Choveau et al., 2011). The reverse voltage-gated mode of HCN2 channel makes the comparison of HCN2 with KCNQ1 fairly difficult. Let us first briefly describe the reverse voltage gating of the HCN2 channel. It has been hypothesized that depolarization of HCN channels uncouples the channel pore from VSDs, which leads to the channel closure (Shin et al., 2004). According to this hypothesis, at hyperpolarized potentials the coupling between S4–S5 linker with S6 reestablishes itself resulting in stabilized open state. This assumes, that mutations disabling the association of VSDs with the PD at hyperpolarized potentials will drive the HCN channels into permanently desensitized state, i.e., channels will be closed at all voltages. Mutagenesis of S4–S5 linker and the end of S6 segment of HCN2 channels, however, indicated that the vast majority of mutants were functional (Chen et al., 2001; Decher et al., 2004). Moreover, large number of mutants exhibited constitutively open component. S4–S5 linker mutants Y331 and R339 demonstrated a complete constitutive conductance (Chen et al., 2001). Therefore, Sanguinetti and colleagues suggested that a significant reduction of the coupling strength in HCN2 is a likely reason for partial or complete constitutive conductance of mutant channels (Chen et al., 2001; Decher et al., 2004). Thus, the mechanism of HCN2 gating is apparently more complex than it is originally thought. A systematic analysis of the voltage-sensor movement in mutant channels by measuring the gating current or fluorescence properties of labeled residues can help us to reveal more about the role of coupling in HCN2 channel gating in future. Nevertheless, since a large number of mutant HCN2 channels are characterized in the literature, we were interested whether the fraction of constitutive conductance is dependent on voltage-gated properties of HCN2 channel as we previously revealed for KCNQ1.

## Constitutive Conductance in KCNQ1 and HCN2 is Linked to Voltage-Dependent Closed-Open Equilibrium of the Pore

**Scheme 1:**
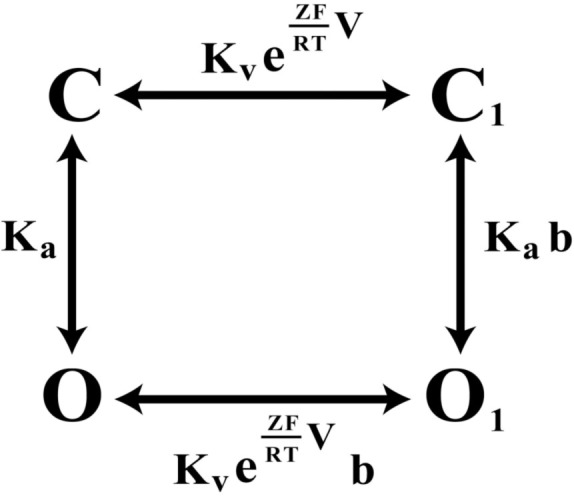
**The four-state allosteric gating scheme, illustrating the voltage-dependent (horizontal) and voltage-independent (vertical) channel transitions**. C and C1 signify the closed-states of the channel, O and O1 the open states, correspondingly.

Large-scale mutagenesis of S4–S5 linker and PD residues of KCNQ1 revealed a strong correlation between the fraction of constitutive conductance (*G*_min_) and the midpoint of the voltage-dependent conductance (*V*_1/2_; Ma et al., 2011). The *V*_1/2_–*G*_min_ correlation in KCNQ1 could be well described by an Eq. 1, which is based on a simple allosteric gating scheme (Scheme 1) involving a voltage-dependent and a voltage-independent closed to open transition as shown below (Ma et al., 2011).

Kv·exp(*ZFV*/*RT*) component describes the voltage-dependent equilibrium constant. *Z*, *F*, and *R* have their usual meanings, *T* is the absolute temperature; *K*_a_ is equilibrium constant of voltage-independent transitions; *b* is an allosteric factor. The mathematical relationship between constitutively open component – *G*_min_ and the midpoint of potential-sensitive fraction – *V*_1/2_ is given by equation:

(1) $$G_{\text{min}}=\frac{1+Kv\cdot e^{\frac{ZF}{RT}\cdot V_{\text{1/2}}}}{\left(b-1\right)\cdot Kv\cdot e^{\frac{ZF}{RT}\cdot V_{\text{1/2}}}}$$

**Figure 3:**
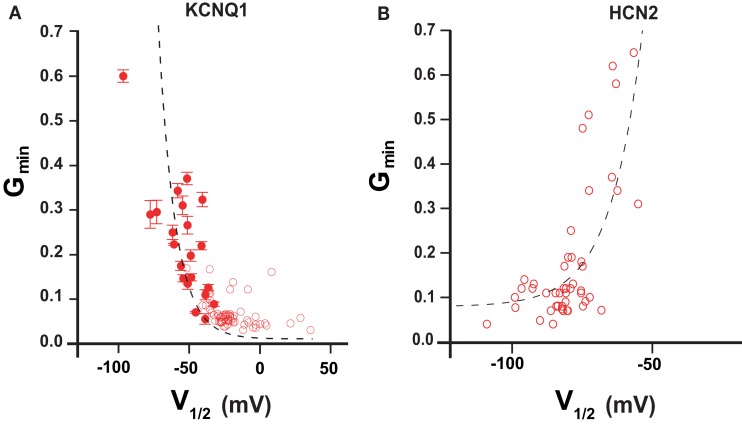
***G*_min_–*V*_1/2_ relationship in KCNQ1 and HCN2 channels**. **(A)** *V*_1/2_–*G*_min_ correlation according to mutation-induced gating perturbations analysis of KCNQ1 (Ma et al., 2011) **(B)** *V*_1/2_–*G*_min_ correlation for HCN2 channel calculated from published studies (Chen et al., 2001; Decher et al., 2004). Mutant channels showing *G*_min_ values larger than 0.7 are omitted (see restriction *G*_min_ <1; Ma et al., 2011). Dashed lines correspond to a theoretical calculation based on a simple allosteric gating scheme proposed earlier (Ma et al., 2011). Parameters are as follows: HCN2 Kv = 1.2 × 10^−3^; Z = 2.27; *b* = 13.6, KCNQ1 Kv = 4.2 × 10^−2^; *Z* = 2.37; *b* = 1.59 × 10^3^.

We were interested whether the *G*_min_–*V*_1/2_ correlation observed with KCNQ1 mutants might be seen also for HCN2 mutants. The estimation method for constitutive conductance component in HCN2 mutants was the same as we previously described for KCNQ1 (Ma et al., 2011). Analyzing mutagenesis data of HCN2 channel (Chen et al., 2001; Decher et al., 2004) we observed that many mutations in HCN2 induce large shifts in GV relationship. Next we plotted *G*_min_ versus *V*_1/2_ for HCN2 channel. The resulting *G*_min_–*V*_1/2_ correlation was well described quantitatively by the allosteric gating model developed for KCNQ1 (Figure 3). The analysis indicates that KCNQ1 and HCN2 channels share a common feature – the equilibrium between voltage-dependent and voltage-independent transitions can be characterized by parameter **b**.

Unfortunately, the quantity of available mutational data for hERG channel was insufficient for a *V*_1/2_–*G*_min_ correlation analysis. It is, however, noteworthy that hERG-bEAG chimeras with negatively shifted *V*_1/2_ parameter also demonstrate an increase in *G*_min_ (Ferrer et al., 2006). In contrast, *Shaker* mutants remain tightly closed at hyperpolarized potentials (Yifrach and MacKinnon, 2002; Soler-Llavina et al., 2006; Ma et al., 2011) in spite of large shifts in their GV curve toward more negative potentials. Exceptions are P475 mutations that we discussed above.

A small constitutively conducting component was observed in single channel recordings of wild type HCN2 and BK channels (Gauss et al., 1998; Chen et al., 2001; Ma et al., 2011). The constitutive conduction in these channels reflects spontaneous fluctuations between open and closed states, which are observable even at the absence of their respective ligands (Proenza and Yellen, 2006; Yang et al., 2010). Earlier reports of constitutive association of the Ca^2+^-binding protein calmodulin (CaM) with the C-terminus of KCNQ1 (Yus-Najera et al., 2002; Ghosh et al., 2006; Shamgar et al., 2006; Ciampa et al., 2011) prompted us to consider intracellular Ca^2+^ as a putative ligand for KCNQ1-CaM complex. Our preliminary data indicate that Ca^2+^ could act as an allosteric effector for KCNQ1 channel. The data are analogous to what has been observed for HCN2 (Proenza and Yellen, 2006; Biel et al., 2009; Kusch et al., 2010) and BK channels (Horrigan and Aldrich, 2002; Biel et al., 2009; Lee and Cui, 2010). In this context, it is of note that a recent analysis of VSD-movement of KCNQ1 suggested that the channel gating follows to the allosteric mechanism (Osteen et al., 2012).

Taken together, our analysis suggests that depending on channel type, the electromechanical coupling strength differentially influences the gating properties of ion channels. Particularly, if the channel pore prefers the closed state in absence of VSDs, as in case of *Shaker*, uncoupling would lead a tightly closed pore. Conversely, if the open state of channel pore is intrinsically more stable, uncoupling would lead to channels with partial or complete constitutively conductance. This seems to be the case for voltage-gated channels that possess C-terminal ligand-binding domains, e.g., KCNQ1, HCN2, and BK channels. From this point of view ligand-binding domains could be seen as “additional” gating machineries attached to the C-terminal part of S6 in HCN2, BK, and KCNQ1 channels that significantly influence the intrinsic pore properties. Consistent with this idea is the observation that the frequency of spontaneous openings in BK channels at resting state are dependent on the length of C-linker connecting S6 segment to RCK domains (Niu et al., 2004). Interestingly, the recent analysis of BK channel pore by cysteine modification experiments revealed that the inner BK channel pore at rest is significantly larger than the one in *Shaker* (Zhou et al., 2011). On the other hand modulation of channel gating by ligand-binding is likely to occur only if coupling between VSDs and the gate is not as dominant as in *Shaker*. Weaker coupling strength would enables fine-tuning of voltage-activated ion channels by their corresponding ligands.

## Future Perspectives

Finding answers to the following two conceptually important questions will significantly broaden our understanding about electromechanical coupling in voltage-gated ion channels.

1) What molecular forces determine the stability of the closed state in *Shaker* and in other potassium channels? An important step toward answering this question is the determination of closed Kv channel structures at defined membrane potential. Ideally, one may investigate channel structure in a distinct membranous environment at different membrane potentials in order to follow the activation process *in situ*. This seems particularly important in the light of recent observations suggesting that in some Kv channels interaction of S4–S5 linker with the distal S6 is dependent on channel state (Grabe et al., 2007; Barghaan and Bahring, 2009; Choveau et al., 2011). It is noteworthy that currently available crystal structures of Kv channels were obtained in a non-membranous environment, i.e., in the absence of an electric field (Long et al., 2005a, 2007). Though it is unclear what kind of channel state is generated under the conditions of crystallization, it can be argued that the conditions drive VSDs into a state described as closed-relaxed state (Villalba-Galea et al., 2008; Jensen et al., 2012). However, further structural and functional studies are required to correlate functional channel state with appropriate structure.
2) Which state is the thermodynamically preferred state of the pore in the absence of voltage-sensors in *Shaker* and in distantly related ion channels? Finding a clear-cut answer to this question is important for understanding the nature of constitutive conductance of ion channels.

Extending the scanning mutagenesis studies to the single channel level will allow a more profound evaluation of coupling properties. Single channel recordings will provide us with valuable information on single channel conductance, duration of closed and opened states, maximal open probability and open probability at hyperpolarized potentials. Ideally, single channel measurements can be combined with structural studies (Sonnleitner et al., 2002). Applying novel techniques, such as solid-state NMR spectroscopy on Kv channels will certainly provide answers as well as raise new questions about the molecular mechanisms underling the electromechanical coupling and about its role in shaping voltage-gated properties of ion channels.

## Conflict of Interest Statement

The authors declare that the research was conducted in the absence of any commercial or financial relationships that could be construed as a potential conflict of interest.
